# Fractional-Order Deep Backpropagation Neural Network

**DOI:** 10.1155/2018/7361628

**Published:** 2018-07-03

**Authors:** Chunhui Bao, Yifei Pu, Yi Zhang

**Affiliations:** College of Computer Science, Sichuan University, Chengdu 610065, China

## Abstract

In recent years, the research of artificial neural networks based on fractional calculus has attracted much attention. In this paper, we proposed a fractional-order deep backpropagation (BP) neural network model with *L*_2_ regularization. The proposed network was optimized by the fractional gradient descent method with Caputo derivative. We also illustrated the necessary conditions for the convergence of the proposed network. The influence of *L*_2_ regularization on the convergence was analyzed with the fractional-order variational method. The experiments have been performed on the MNIST dataset to demonstrate that the proposed network was deterministically convergent and can effectively avoid overfitting.

## 1. Introduction

It is well known that artificial neural networks (ANNs) are the abstraction, simplification, and simulation of the human brains and reflect the basic characteristics of the human brains [1]. In recent years, great progress has been made in the research of deep neural networks. Due to the powerful ability of complex nonlinear mapping, many practical problems have been successfully solved by ANNs in the fields of pattern recognition, intelligent robot, automatic control, prediction, biology, medicine, economics, and other fields [2, 3]. BP neural network is one of the most basic and typical multilayer forward neural networks, which are trained by backpropagation (BP) algorithm. BP, which is an efficient way for optimization of ANNs, was firstly introduced by Werbos in 1974. Then, Rumelhart and McCelland et al. implemented the BP algorithm in detail in 1987 and applied it to the multilayer network version of Minsky [4–6].

The fractional calculus has a history as long as the integral order calculus. In the past three hundred years, the theory of fractional calculus has made great progresses [7–11]. Its basics are differentiation and integration of arbitrary fractional order. Nowadays, fractional calculus is widely used in diffusion processes [12–14], viscoelasticity theory [15], automation control [16–18], signal processing [19–21], image processing [22–25], medical imaging [26–28], neural networks [29–37], and many other fields. Due to the long-term memory, nonlocality, and weak singularity characteristics [29–37], fractional calculus has been successfully applied to ANNs. For instance, Boroomand constructed the Hopfield neural networks based on fractional calculus [37]. Kaslik analyzed the stability of Hopfield neural networks [30]. Pu proposed a fractional steepest descent approach and offered a detailed analysis of its learning conditions, stability, and convergence [38]. Wang applied the fractional steepest descent algorithm to train BP neural networks and proved the monotonicity and convergence of a three-layer example [33]. However, there are three limitations in the proposed fractional-order BP neural network models in [33]. First, the neural network in [33] just had 3 layers, which was actually a shadow network and was not proper to demonstrate its potential for deep learning. Second, the fractional order *v* of this model was restricted to (0,1] without reasonable analysis. Third, the loss function did not contain the regularization term, which is an efficient way to avoid overfitting, especially when the training set has small scalar. Overfitting means that the model has high prediction accuracy on training set but has the low prediction accuracy on testing set. This makes the generalization ability of the model poor, and the application value is greatly reduced.

In this paper, we proposed a deep fractional-order BP neural network with *L*_2_ regularization term, and the fractional-order *v* could be any positive real number. With the fractional-order variational method, the influence of *L*_2_ regularization on the convergence of the proposed model was exploited. The performance of the proposed model was evaluated on the MINST dataset.

The structure of the paper is as follows: in Section 2, the definitions and simple properties of fractional calculus are introduced. In Section 3, the proposed fractional-order multilayer BP neural networks are given in detail. In Section 4, the necessary conditions and the influence of *L*_2_ regularization for the convergence of the proposed BP algorithm are stated. In Section 5, experimental results are presented to illustrate the effectiveness of our model. Finally, the paper is concluded in Section 6.

## 2. Background Theory for Fractional Calculus

In this section, the basic knowledge of fractional calculus is introduced, including the definitions and several simple properties used in this paper.

Different from integer calculus, fractional derivative does not have a unified temporal definition expression up to now. The commonly used definitions of fractional derivative are Grünwald-Letnikov (G-L), Riemann-Liouville (R-L), and Caputo derivatives [7–11].

The following is the G-L definition of fractional derivative:

(1) DxvaG−Lfx≜limh→0⁡ h−v∑k=0x−a/h−vkfx−kh≜limN→∞⁡x−a/N−vΓ−v·∑k=0N−1Γk−vΓk+1fx−kx−aN

where

(2) $$\begin{array}{c} \left(\begin{array}{c} -v\\ k \end{array}\right)=\frac{\left(-v\right)\left(-v+1\right)\ldots \left(-v+k-1\right)}{k!} \end{array}$$

_*a*_^*G* − *L*^*D*_*x*_^*v*^ denotes the fractional differential operator based on G-L definition, *f*(*x*) denotes a differintegrable function, *v* is the fractional order, [*a*, *x*] is the domain of *f*(*x*), Γ is the Gamma function, and [·] is the rounding function.

The R-L definition of fractional derivative is as follows:

(3) DxvaR−Lfx=1Γn−vdndxn∫axfyx−yv−n+1dy

where _*a*_^*R* − *L*^*D*_*x*_^*v*^ denotes the fractional differential operator based on G-L definition; *n* = [*v* + 1]. Moreover, the G-L fractional derivative can be deduced from the definition of the R-L fractional derivative.

The Caputo definition of fractional derivative is as follows:

(4) DxvaCfx=1Γn−v∫axx−yn−v−1fnydy

where _*a*_^*C*^*D*_*x*_^*v*^ is the fractional differential operator based on Caputo definition, *n* = [*v* + 1].

Fractional calculus is more difficult to compute than integer calculus. Several mathematical properties used in this paper are given here. The fractional differential of a linear combination of differintegral functions is as follows:

(5) $$\begin{array}{c} D_{x}^{v}\left(\;\lambda f\left(\;x\;\right)+\beta g\left(\;x\;\right)\;\right)=\lambda D_{x}^{v}f\left(\;x\;\right)+\beta D_{x}^{v}g\left(\;x\;\right) \end{array}$$

where *f*(*x*) and *g*(*x*) are differintegral functions and *λ* and *β* are constants.

The fractional differential of constant function *f*(*x*) = C, (C is a constant) is different under different definitions:

For the G-L definition,

(6) DxvaG−Lfx=limN→∞⁡x−a/N−vΓ−v∑k=0N−1Γk−vΓk+1C=Cx−a−vΓ1−v

For the R-L definition,

(7) DxvaR−Lfx=Cx−a−vΓ1−v,v>0

And for the Caputo definition

(8) DxvaCfx=0,v>0

According to (6), (7) and (8), we can know that for the G-L and R-L definition, the fractional differential of constant function is not equal to 0. Only with the Caputo definition, the fractional differential of constant function equals to 0, which is consistent to the integer-order calculus. Therefore, the Caputo definition is widely used in solving engineering problems and it was employed to calculate the fractional-order derivative in this paper. The fractional differential of function *f*(*x*) = (*x* − *a*)^*p*^, (*p* > −1) is as follows:

(9) $$\begin{array}{c} \frac{d^{v}{\left(x-a\right)}^{p}}{{dx}^{v}}=\frac{\Gamma \left(p+1\right){\left(x-a\right)}^{p-v}}{\Gamma \left(p-v+1\right)} \end{array}$$

## 3. Algorithm Description

### 3.1. Fractional-Order Deep BP Neural Networks

In this section, we introduce the fractional-order deep BP neural network with L layers. *n*^*l*^, *l* = 1,2,…, L, is the number of neurons for the *l*-th layer. W^*l*^ = (*w*_*ji*_^*l*^)_*n*^*l*+1^×*n*^*l*^_ denotes the weight matrix connecting the *l*-th layer and the (*l* + 1)-th layer. *f*_*l*_ denotes the corresponding activation function for the *l*-th layer. *X*^*j*^ and *O*^*j*^ are the input and the corresponding ideal output of the *j*-th sample and the training sample set is {*X*^*j*^, *O*^*j*^}_*j*=1_^*J*^. *Z*^*l*^ = (*z*_1_^*l*^, *z*_2_^*l*^ …, *z*_*n*^*l*+1^_^*l*^) denotes the total inputs of *l*-th layer. If neurons in the *l*-th layer are not connected to any neurons in previous layer, these neurons are called external outputs of the *l*-th layer, denoted as *A*_1_^*l*^. On the contrary, if neurons in the *l*-th layer are connected to every neuron in previous layer, these neurons are called internal outputs of *l*-th layer, denoted as *A*_2_^*l*^. *A*^*l*^ = (*a*_1_^*l*^, *a*_2_^*l*^ …, *a*_*n*^*l*^_^*l*^) denotes the total outputs of *l*-th layer. The forward computing of the fractional-order deep BP neural networks is as follows:

(10) A2l=flZl

(11) Al=A1lA2l

(12) Zl+1=Wl·Al

Particularly, external outputs can exist in any layer except the last one. With the square error function, the error corresponding to *j*-th sample can be denoted as:

(13) $$\begin{array}{c} E_{j}=\frac{1}{2}{\left\|\;A_{j}^{L}-O_{j}\;\right\|}^{2}=\frac{1}{2}\overset{n^{L}}{\underset{i=1}{\sum }}{\left(\;a_{ji}^{L}-o_{ji}\;\right)}^{2} \end{array}$$

where *a*_*ji*_^*L*^ denotes the *i*-th element of *A*_*j*_^*L*^, *o*_*ji*_ denotes the *i*-th element of *O*_*j*_.

The total error of the neural networks is defined as

(14) $$\begin{array}{c} E=\overset{J}{\underset{j=1}{\sum }}E_{j}=\frac{1}{2}\overset{J}{\underset{j=1}{\sum }}{\left\|\;A_{j}^{L}-O_{j}\;\right\|}^{2}=\frac{1}{2}\overset{J}{\underset{j=1}{\sum }}\overset{n^{L}}{\underset{i=1}{\sum }}{\left(\;a_{ji}^{L}-o_{ji}\;\right)}^{2}. \end{array}$$

In order to minimize the total error of the fractional-order deep BP neural network, the weights are updated by the fractional gradient descent method with Caputo derivative. Let *i* = 1,2, ..., *n*^*l*^. The backpropagation of fractional-order deep BP neural networks can be derived with the following steps.

Firstly, we define that

(15) $$\begin{array}{c} \delta_{i}^{l}=\frac{\partial E}{\partial z_{i}^{l}}. \end{array}$$

According to (13), we can know that

(16) $$\begin{array}{c} \delta_{i}^{L}=\frac{\partial E}{\partial z_{i}^{L}}=\overset{J}{\underset{j=1}{\sum }}\left(\;a_{ji}^{L}-o_{ji}\;\right)f_{L}^{\prime }\left(\;z_{i}^{L}\;\right). \end{array}$$

Then the relationship between *δ*_*i*_^*l*^ and *δ*_*i*_^*l*+1^ can be given by

(17) δil=∂E∂zil=∑j=1nl+1∂E∂zjl+1∂zjl+1∂zil=∑j=1nl+1δjl+1·wjilfl′zil=fl′zil∑j=1nl+1δjl+1·wjil.

Then, according to the chain rule and (17), we have

(18) $$\begin{array}{c} D_{w_{ji}^{l}}^{v}E=\frac{\partial E}{\partial z_{j}^{l+1}}\cdot D_{w_{ji}^{l}}^{v}z_{j}^{l+1}=\delta_{j}^{l+1}\cdot a_{i}^{l}\cdot \frac{{\left(w_{ji}^{l}\right)}^{1-v}}{\Gamma \left(2-v\right)}. \end{array}$$

The updating formula is

(19) $$\begin{array}{c} {\left(\;w_{ji}^{l}\;\right)}^{t+1}={\left(\;w_{ji}^{l}\;\right)}^{t}-\eta D_{{w_{ji}^{l}}^{t}}^{v}E \end{array}$$

where *t* ∈ N denotes the *t*-th iteration and *η* > 0 is the learning rate.

### 3.2. Fractional Deep BP Neural Networks with *L*_2_ Regularization

Fractional-order BP neural network can be overfitted easily when the training set has small scalar. *L*_2_ regularization is a useful way to avoid models to be overfitted without modifying the architecture of network. Therefore, by introducing the *L*_2_ regularization term into the total error, the modified error function can be presented as

(20) $$\begin{array}{c} E_{L2}=E+\frac{\lambda }{2}{\left\|\;W\;\right\|}^{2} \end{array}$$

where ‖*W*‖^2^ denotes the sum of squares of all weights and *λ* ≥ 0 denotes the regularization parameter.

By introducing (18), we have

(21) $$\begin{array}{c} D_{w_{ji}^{l}}^{v}E_{L2}=D_{w_{ji}^{l}}^{v}E+\lambda \frac{{\left(w_{ji}^{l}\right)}^{2-v}}{\Gamma \left(3-v\right)}. \end{array}$$

The updating formula is

(22) $$\begin{array}{c} {\left(\;w_{ji}^{l}\;\right)}^{t+1}={\left(\;w_{ji}^{l}\;\right)}^{t}-\eta D_{{\left(w_{ji}^{l}\right)}^{t}}^{v}E_{L2} \end{array}$$

where *t* ∈ N denotes the *t*-th iteration and *η* > 0 is the learning rate.

## 4. Convergence Analysis

In this section, the convergence of the proposed fractional-order BP neural network is analyzed. According to previous studies [39–42], there are four necessary conditions for the convergence of BP neural networks:

(1) The activation functions *f*_*l*_, (*l* = 1,2,…, *L*) are bounded and infinitely differentiable on R and all of their corresponding derivatives are also continuous and bounded on *R*. This condition can be easily satisfied because the most common sigmoid activation functions are uniformly bounded on *R* and infinitely differentiable.

(2) The boundedness of the weight sequence {(*w*_*ji*_^*l*^)^*t*^} is valid during training procedure and *Ω* ∈ *R*^∑_1_^*L*−1^*n*^*l*^·*n*^*l*+1^^ is the domain of all weights with certain boundary.

(3) The learning rate *η* > 0 has an upper bound.

(4) Let *W* denote the weights matrix that consists of all weights and *ϕ* = {*W*∣*D*_*W*_^*v*^*E*_*L*2_ = 0} be the *v*-order stationary point set of the error function. One necessary condition is that *ϕ* is a finite set.

Then, the influence of *L*_2_ regularization on the convergence is derived by using the fractional-order variational method.

According to (20), *E*_*L*2_ is defined as a fractional-order multivariable function. The proposed fractional-order BP algorithm is to minimize *E*_*L*2_. Let *U* denote the fractional-order extreme point of *E*_*L*2_ and *ξ* denotes an admissible point. In addition, *U* is composed of *U*^1^, *U*^2^,…, *U*^*L*−1^ where *U*^*l*^  (*l* = 1,2,…, *L* − 1) denotes the weights matrix between the *l*-th and (*l* + 1)-th layer when *E*_*L*2_ reaches the extreme value. *ξ* is composed of *ξ*^1^, *ξ*^2^,…, *ξ*^*L*−1^ where *ξ*^*l*^ corresponds to *U*^*l*^. The initial weights are random values, so the initial points of weights can be represented as *U* + (*α* − 1)*ξ*, where *α* is a vector that consists of small parameters *α*_1_, *α*_2_,…, *α*_*L*−1_, and *α*_*l*_ corresponds to *U*^*l*^ and *ξ*^*l*^. If *α* = 1, it means *α*_*l*_ = 1(*l* = 1,2,…, *L* − 1), then *U* + (*α* − 1)*ξ* = *U*, and *E*_*L*2_ reaches the extreme value. Thus, the process of training the BP neural networks from a random initial weight *W* to U can be treated as the process of training *α* with a random initial value to *α* = 1.

The fractional-order derivative of *E*_*L*2_ on *U* + (*α* − 1)*ξ* is given as

(23) DαvδEL2α=1=Dαvδ1α+δ2αα=1=DαvEU+α−1ξ+Dαvλ2U+α−1ξ2α=1=0

where *v* is the fractional order, which is a positive real number.

From (23), we can see that when *α* = 1, if the *v*-order differential of *E*(*U* + (*α* − 1)*ξ*) with respect to *α* is existent, *δ*_1_(*α*) has a *v*-order extreme point and we have

(24) $$\begin{array}{c} {\left.\;D_{\alpha }^{v}\delta_{1}\left(\;\alpha \;\right)\;\right|}_{\alpha =1}={\left.\;D_{\alpha }^{v}E\left(\;U+\left(\;\alpha -1\;\right)\xi \;\right)\;\right|}_{\alpha =1}=0. \end{array}$$

In this case, the output of each layer in the neural networks is still given by (10) and (11) and the input of each layer is turned into the following:

(25) $$\begin{array}{c} Z^{l+1}=\left(\;U^{l}+\left(\;\alpha_{l}-1\;\right)\xi^{l}\;\right)\cdot A^{l}. \end{array}$$

When *α*_*l*_ = 1, we have

(26) $$\begin{array}{c} Z^{l+1}=U^{l}\cdot A^{l}. \end{array}$$

Without loss of generality, according to (18), for the *l*-th layer of the networks, the *v*-order differential of *E* with respect to *α*_*l*_ can be calculated as

(27) DαlvEUl+αl−1ξlαl=1=∂E∂Zl+1·DαlvZl+1αl=1=δl+1·AlTξlαl1−vΓ2−vαl=1=ξlδl+1·AlTΓ2−v=0.

where *δ*^*l*^ denotes the column vector *D*_*Z*^*l*^_^1^*E*.

Since the value of *ξ* is stochastic, according to variation principle [43], to allow (24) to be set up, a necessary condition is that for every layer of the networks

(28) $$\begin{array}{c} \frac{\left(\delta^{l+1}\cdot {\left(A^{l}\right)}^{T}\right)}{\Gamma \left(2-v\right)}=0. \end{array}$$

Secondly, without loss of generality, for *δ*_2_(*α*) we have

(29) Dαlvδ2αlαl=1=Dαlvλ2Ul+αl−1ξl2αl=1=∑λUjilξjilΓ2−v+λξjil2Γ3−v−λξjil2Γ2−v=λΓ2−vΓ3−v∑UjilξjilΓ3−v+ξjil2Γ2−v−ξjil2Γ3−v=0

To allow (29) to be set up, a necessary condition is

(30) $$\begin{array}{c} \frac{\lambda }{\Gamma \left(2-v\right)\Gamma \left(3-v\right)}=0. \end{array}$$

With (28) and (30), the Euler-Lagrange equation of *D*_*α*_^*v*^*δE*_*L*2_|_*α*=1_ can be written as

(31) $$\begin{array}{c} \frac{\left(\delta^{l+1}\cdot {\left(A^{l}\right)}^{T}\right)}{\Gamma \left(2-v\right)}+\frac{\lambda }{\Gamma \left(2-v\right)\Gamma \left(3-v\right)}=0. \end{array}$$

Equation (31) is the necessary condition for the convergence of the proposed fractional-order BP neural networks with *L*_2_ regularization. From (31), we can see that if *λ* > 0, then (*δ*^*l*+1^ · (*A*^*l*^)^*T*^) ≠ 0. (*δ*^*l*+1^ · (*A*^*l*^)^*T*^) is the first-order derivative of *E* in terms of *U* and can be calculated by *U* and input sample *X*. It means that the extreme point U of the proposed algorithm is not equal to the extreme point of integer-order BP algorithm or fractional-order BP algorithm. *U* changes with the different value of *λ* and *v*. In addition, it is also clear that the regularization parameter *λ* is bounded since the values of input samples *X* and weights *W* are bounded and *v* is a constant during the training process.

## 5. Experiments

In this section, the following simulations were carried out to evaluate the performance of the presented algorithm. The simulations have been performed on the MNIST handwritten digital dataset. Each digit in the dataset is a 28 × 28 image. Each image is associated with a label from 0 to 9. We divided each image into four parts, which were top-left, bottom-left, bottom-right, and top-right, and each part was a 14 × 14 matrix. We vectorized each part of the image as a 196 × 1 vector and each label as a 10 × 1 vector.

**Figure 1:**
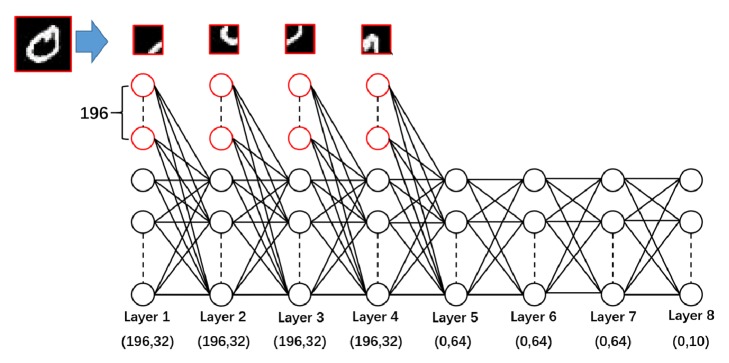
The topological structure of the neural networks.

In order to identify the handwritten digits in MNIST dataset, a neural network with 8 layers was proposed.  Figure 1 shows the topological structure of the neural networks. For the first four layers of the network, each layer has 196 external neurons and 32 internal neurons. The outputs of the external neurons are in turn four parts of an image and the outputs of the internal neurons of the first layer are 1. The last four layers have no external neurons. The fifth layer, sixth layer, and seventh layer have 64 internal nodes and the output layer has ten nodes. The activation functions of all neurons except the first layer are sigmoid functions, which can be given as follows:

(32) $$\begin{array}{c} f\left(\;x\;\right)=\frac{1}{1+e^{-x}}. \end{array}$$

The MNIST dataset has a total number of 60000 training samples and 10000 testing samples. The simulations demonstrate the performance of the proposed fractional-order BP neural network with *L*_2_ regularization, fractional-order BP neural network, traditional BP neural network, and traditional BP neural network with *L*_2_ regularization. To evaluate the robustness of our proposed network for a small set of training samples, we set the number of training samples to be (10000, 20000, 30000, 40000, 50000, and 60000). Different fractional *v*-order derivatives were employed to compute the gradient of error function, where *v* = 1/9, 2/9, 3/9, 4/9, 5/9, 6/9, 7/9, 8/9, 9/9, 10/9, 11/9, 12/9, 13/9, 14/9, 15/9, 16/9, 17/9, 19/9, and 20/9 separately (*v* = 9/9 = 1 corresponds to standard integer-order derivative for the common BP; *v* ≠ 2 because if *v* = 2 the change of weights after each iteration is 0, and the weights of the neural networks cannot be updated). The learning rate was set to be 3 and the batch size was set to be 100. The number of epochs *n* was 300. Two main metrics—training accuracy and testing accuracy—were used to measure the performance of the results from different networks. Each network was trained 5 times and the average values were calculated.

**Figure 2:**
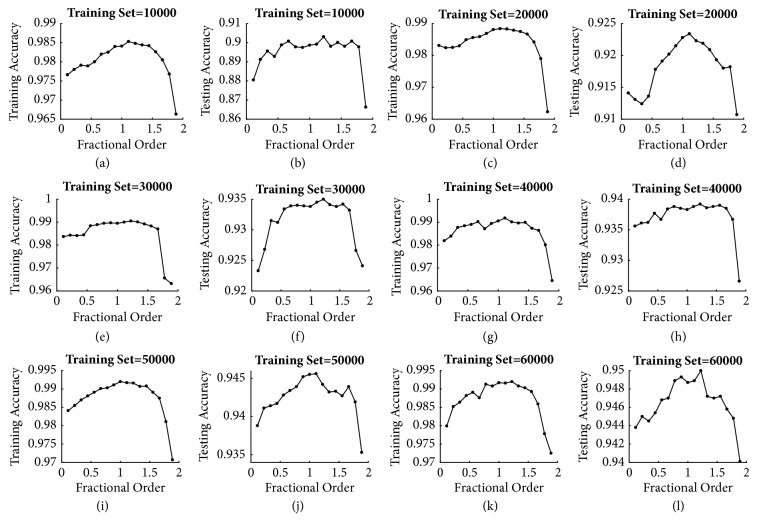
The relationship between the fractional order of gradient descent method and the neural network performance.

In order to explore the relationship between the fractional orders and the neural network performance, the fractional-order neural networks with different orders were trained.  Figure 2 shows the results of different networks with different sizes of training set. We can find that when the fractional order *v* exceeds 1.6, both the training and testing accuracies declined rapidly, and when the fractional order *v* > 2, the performances of the fractional BP neural networks were much poorer than that with 0 < *v* < 2. The results of *v* = 19/9 and 20/9 were shown in Table 1 as examples. This result is consistent with that for describing physical problems, and usually the limitation 0 < *v* < 2 is adopted in the fractional-order models.

From Figure 2, it can be observed that, with the increase of the size of training set, the performances of the networks were improved visibly. Furthermore, it is also obvious that the training and testing accuracies raised gradually with increasing fractional orders and then reached the peak while *v* equaled 10/9 or 11/9 order. After that, the training and testing accuracies began to decline rapidly.

Table 2 shows the optimal orders under training set and testing set separately with different size of training set and it can be noticed that the optimal orders almost concentrated in 10/9 and 11/9. The only exception is that when the number of training samples was 50000, the training accuracy of order 1 was slightly higher than that in 10/9 or 11/9 order case. Generally, for the MNIST dataset the performances of fractional-order BP neural networks are better than integer order.

It also can be seen that, in each case, the training accuracy is much bigger than testing accuracy, which means that the BP neural networks have obvious overfitting phenomenon. To avoid overfitting, the integer-order and fractional-order BP neural networks with *L*_2_ regularization were trained. With different sizes of training set we chose the regularization parameter *λ* to be (2 × 10^−5^, 1 × 10^−5^, 5 × 10^−6^, 5 × 10^−6^, 5 × 10^−6^, and 3 × 10^−6^). For the fractional-order neural networks, we chose the fractional order *v* that had highest testing accuracy in previous simulations. When the numbers of training samples were (10000, 20000, 30000, 40000, 50000, and 60000), we separately set the fractional order *v* to be (11/9, 10/9, 11/9, 11/9, 10/9, 11/9).

**Figure 3:**
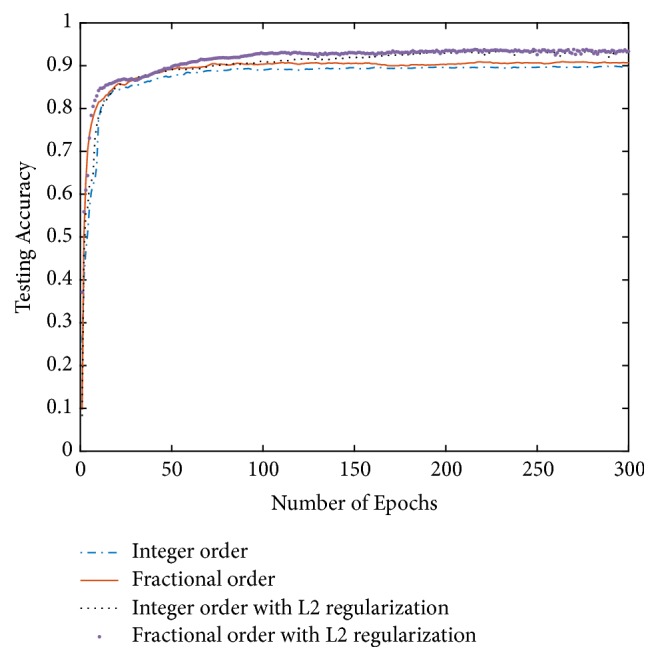
Performance comparison in terms of testing accuracy.

The performance of the proposed fractional-order BP neural networks with *L*_2_ regularization and the performance comparison with integer-order BP neural networks (IOBP), integer-order BP neural networks with *L*_2_ regularization, and fractional-order BP neural networks (FOBP) in terms of training and testing accuracy are shown in Table 3 and the change of the testing accuracy with the iterations was given in Figure 3

In Table 3 and Figure 3, it can be seen that, after the addition of *L*_2_ regularization to BP neural networks, the training accuracy is slightly decreased but the testing accuracy significantly increased, which indicated that adding *L*_2_ regularization can effectively suppress overfitting and improve the generalization of BP neural networks. Furthermore, it can be noticed that after adding *L*_2_ regularization the performance of fractional-order BP neural network is better than integer order. One important merit of the *L*_2_ regularization is that it gained more benefit while the training set is small. The most possible reason is that the network trained with the smallest number of training samples was affected most by the overfitting. With the increase of the training samples, the model gradually changed from overfitting to underfitting, so the improvement of the regularization method became faint.

**Figure 4:**
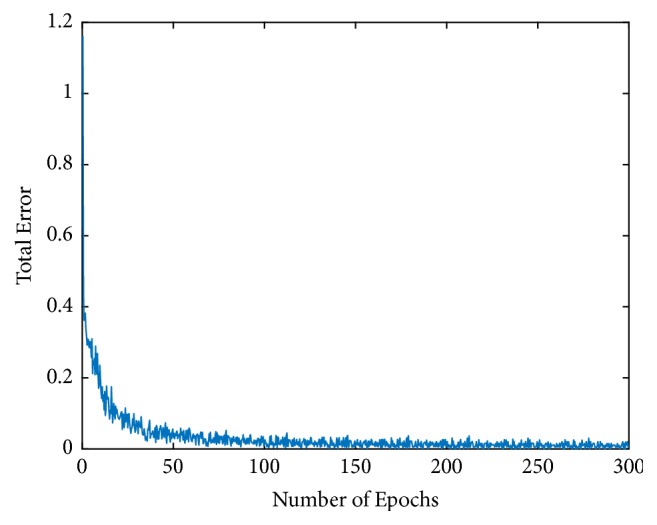
Changes of total error *E*_*L*2_ during the training process.

**Figure 5:**
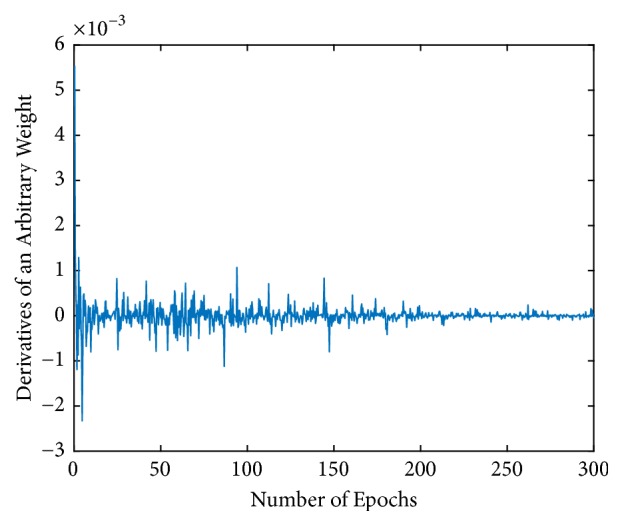
Changes of *D*_*w*_20,20_^5^_^*v*^*E*_*L*2_ during the training process.

Then, the stability and convergence of the proposed fractional-order BP neural networks with *L*_2_ regularization are demonstrated in Figures 4 and 5. We used the network with optimal order, which means that the size of training set was 60000, fractional-order *v* was 11/9, and the regularization parameter *λ* was 3 × 10^−6^.  Figure 4 shows the change of the total error *E*_*L*2_ during the training process. Without loss of generality, the change of *D*_*w*_20,20_^5^_^*v*^*E*_*L*2_ was randomly selected and Figure 5 shows the change of it during the training process. It is clear to see that *E*_*L*2_ and *D*_w_^*v*^*E*_*L*2_ converged fast and stably and were finally close to zero. These observations effectively verify the proposed algorithm is deterministically convergent.

## 6. Conclusion

In this paper, we applied fractional calculus and regularization method to deep BP neural networks. Different from previous studies, the proposed model had no limitations on the number of layers and the fractional-order was extended to arbitrary real number bigger than 0. *L*_2_ regularization was also imposed into the errorless function. Meanwhile, we analyzed the profits introduced by the *L*_2_ regularization on the convergence of this proposed fractional-order BP network. The numerical results support that the fractional-order BP neural networks with *L*_2_ regularization are deterministically convergent and can effectively avoid the overfitting phenomenon. Then, how to apply fractional calculus to other more complex artificial neural networks is an attracted topic in our future work.

## Figures and Tables

**Table 1:** Performances of the algorithms when v>2.

Size of training set	*v* = 19/9		*v* = 20/9	
	Train Accuracy	Test Accuracy	Train Accuracy	Test Accuracy
10000	88.65%	83.52%	76.31%	72.66%
20000	91.04%	89.52%	78.93%	75.97%
30000	93.03%	90.65%	82.51%	80.79%
40000	93.20%	90.53%	82.47%	80.61%
50000	93.02%	91.23%	82.53%	81.60%
60000	93.85%	91.71%	87.32%	86.05%

**Table 2:** Optimal Orders and Highest Accuracies.

Size of training set	Optimal order of training set	Optimal order of testing set	Highest training accuracy	Highest testing accuracy
10000	10/9	11/9	98.53%	90.31%
20000	10/9	10/9	98.84%	92.34%
30000	11/9	11/9	99.05%	93.50%
40000	10/9	11/9	99.18%	93.92%
50000	1	10/9	99.20%	94.56%
60000	11/9	11/9	99.20%	95.00%

**Table 3:** Performance comparison of different type BP neural networks.

Size of training set	Integer-order BP neural networks		Fractional-order BP neural networks		Integer-order BP neural networks with *L*_2_ regularization		Fractional-order BP neural networks with *L*_2_ regularization			
	Training Accuracy	Testing Accuracy	Training Accuracy	Testing Accuracy	Training Accuracy	Testing Accuracy	Training Accuracy	Testing Accuracy	Improvement relative to IOBP	Improvement relative to FOBP
10000	98.41%	89.87%	98.48%	90.31%	98.45%	93.35%	98.43%	93.95%	4.54%	4.03%
20000	98.81%	92.28%	98.84%	92.34%	98.75%	95.09%	98.79%	95.13%	3.09%	3.02%
30000	98.95%	93.38%	99.05%	93.50%	98.92%	95.15%	98.88%	95.62%	2.40%	2.27%
40000	99.05%	93.83%	99.01%	93.92%	98.96%	95.63%	98.95%	95.83%	2.13%	2.03%
50000	99.20%	94.55%	99.17%	94.56%	99.11%	96.08%	99.15%	96.45%	2.01%	2.00%
60000	99.17%	94.87%	99.20%	95.00%	99.13%	96.51%	99.17%	96.70%	1.93%	1.79%

We use the following formula to calculate improvement: improvement of A compared with B = (A-B)÷B.

## Data Availability

The code of this work can be downloaded at https://github.com/BaoChunhui/Deep-fractional-BP-neural-networks.
